# A novel endothelial-derived anti-inflammatory activity significantly inhibits spontaneous choroidal neovascularisation in a mouse model

**DOI:** 10.1186/s13221-016-0036-4

**Published:** 2016-05-11

**Authors:** Laura Paneghetti, Yin-Shan Eric Ng

**Affiliations:** UCL Institute of Ophthalmology, London, UK; Schepens Eye Research Institute, 20 Staniford Street, Boston, MA 02114 USA; Xeptagen S.p.A., Marghera Venice, Italy

**Keywords:** Endothelium, Inflammation, NF-kB, Intimal hyperplasia, Choroidal neovascularisation

## Abstract

**Background:**

Endothelial cells (EC) grown on collagen particles inhibit intimal hyperplasia in animal models when applied perivascularly, and this effect appears to be, at least in part, the result of EC-derived soluble factors that suppress local vascular inflammation. To elucidate the molecular basis of the therapeutic effects of EC grown on collagen particles, the anti-inflammatory activity of conditioned medium from these cells was characterized.

**Methods:**

Human aortic EC (HAEC) and, for chromatin immunoprecipitation assays, human umbilical vein EC (HUVEC) were treated with tumor necrosis factor alpha (TNFα) in the presence of conditioned medium generated by HAEC grown on collagen particles (ECPCM), and the anti-inflammatory effects were evaluated by analysing the expression of the inflammation-related adhesion molecules E-selectin and vascular cell adhesion molecule-1 (VCAM-1). The therapeutic activity of ECPCM was studied using the mouse strain JR5558, which develops spontaneous choroidal neovascularisation (CNV) lesions driven by local inflammation.

**Results:**

ECPCM significantly suppressed TNFα-induced expression of E-selectin and VCAM-1. ECPCM did not affect the mRNA stability of the two genes, but suppressed TNFα-induced binding of the p65 subunit of NF-kB transcription factor to E-selectin and VCAM-1 promoters. In vivo, systemic ECPCM treatment significantly reduced the CNV area and the recruitment of activated macrophages to the lesions. Characterization of the molecule responsible for the anti-inflammatory activity in ECPCM indicates that it is unlikely to be a protein and that it is not any of the better characterized EC-derived anti-inflammatory molecules.

**Conclusions:**

Medium conditioned by HAEC grown on collagen particles exhibits significant anti-inflammatory activity via inhibition of genes that mediate inflammatory responses in EC.

**Electronic supplementary material:**

The online version of this article (doi:10.1186/s13221-016-0036-4) contains supplementary material, which is available to authorized users.

## Background

As the innermost layer of blood vessels, endothelial cells (EC) regulate vascular physiology by producing a variety of soluble factors and metabolites that affect circulating blood elements and the underlying vascular smooth muscle cells (VSMC). In this way, EC control thrombosis, coagulation, vasomotor tone, blood flow and cell growth [1, 2]. The endothelium also plays a key role in immune and inflammatory reactions by regulating leukocyte adhesion, activation and migration into the tissue [1, 2]. EC are usually in a quiescent, anti-coagulant, non-thrombogenic and non-inflammatory state [2, 3]. However, EC can be readily activated by a variety of stimuli, including cytokines, thrombin, histamine, physical injury and active infections. Once activated, EC express different classes of adhesion molecules that mediate the increased interaction with leukocytes: selectins, such as E-selectin, which bind to carbohydrate determinants on leukocytes to facilitate rolling; members of the immunoglobulin superfamily such as intercellular adhesion molecule-1 (ICAM-1); and vascular cell adhesion molecule-1 (VCAM-1), which binds to integrins to mediate firm adhesion and trans-endothelial migration of leukocytes [2, 4].

An uncontrolled and chronic inflammatory response and discordant stimulation of EC are common events in many pathological processes, including the development of intimal hyperplasia (IH) [1, 5] and choroidal neovascularisation (CNV) [6, 7]. IH is characterized by an increase in the number of cells in the intima. It is a distinctive state of vascular remodelling in which VSMC proliferate and migrate from the medial layer into the intima, accompanied by an increase in the amount of extracellular matrix [8, 9], with a resultant reduction in the vessel lumen diameter and blood flow which may result in occlusion [9, 10]. IH occurs in atherosclerotic vessels, hypertensive pulmonary arteries, venous and prosthetic bypass grafts and as a complication of transluminal angioplasty, stent placement or surgical repair [5, 8, 10]. CNV is characterised by inflammation and the invasion of new and immature vessels from the choriocapillaris thorough the retinal pigmented epithelium at the posterior pole of the eye, and is a major blinding complication associated with age-related macular degeneration (AMD) [6, 11]. Convincing epidemiological data suggest that AMD is associated with an increased risk for cardiovascular disease, including IH; it is possible that uncontrolled vascular inflammation is the common driving factor for these pathologies [12–15].

Promising efforts to treat IH have used a cell-based therapeutic approach in which EC were grown on a three-dimensional (3-D) collagen matrix (EC/matrix) that was surgically placed around or injected perivascularly to angioplasty- or stent-treated vessels of porcine or murine animal models. The animals treated with the EC/matrix showed less restenosis and reduced inflammation compared to control untreated animals [5, 16–18]. Since the EC/matrix was delivered in the perivascular space, at a distance from the media and intima of the affected vessel, a soluble factor (or soluble factors) released from the cultured EC is likely responsible for the beneficial effects.

Based on these observations, conditioned media obtained from EC grown on 3-D collagen particles (ECPCM) was produced and analysed for its anti-inflammatory potential in vitro and in vivo. In this report we show that ECPCM reduced tumor necrosis factor alpha (TNFα)-induced expression of pro-inflammatory adhesion molecules in EC grown on tissue culture plates. This anti-inflammatory effect was mediated by inhibition of the binding by the activated NF-kB transcription factor to the promoters of its target genes. The therapeutic potential of ECPCM was also demonstrated using an animal model of inflammation-driven spontaneous CNV. Our findings provide insight into the homeostatic control exerted by EC grown on 3-D collagen particles over the vascular system and offer a potential therapeutic strategy for the treatment of vascular inflammation and its associated pathologies.

## Methods

### Cell culture

Human aortic endothelial cells (HAEC) were grown in endothelial growth medium 2 (EGM2) and used at passages seven and eight. Human umbilical vein endothelial cells (HUVEC) were grown in EGM2 and used at passages six and seven. For studies of the effect of conditioned medium, HAEC and HUVEC were plated in EGM2 without hydrocortisone and treated using collection medium (phenol red free-endothelial basal medium (EBM), 0.5 % fetal bovine serum (FBS), 50 μg/ml gentamicin) or ECPCM. Cells and growing media were purchased from Lonza (Slough, UK).

### Generation of conditioned medium by EC on collagen particles (ECPCM)

HAEC were seeded on collagen particles purified from porcine skin (Gelfoam® powder, Pfizer, Tadworth, UK) and cultured at 37 °C, 5 % CO_2_ for 15 days with media changes every other day, in accordance with published methods [16, 17]. On day 15 the number of viable cells was determined in duplicated culture to ensure the desirable cell density of >1.8 × 10^6^ cells/tube was reached. For collection of ECPCM, the growing medium was replaced by collection medium and the cells cultured for an additional 24–27 h. ECPCM was collected, passed through a sterile filter with 0.2 μm pore size (Acrodisc® Filter, Pall Corp, Port Washington, NY), aliquoted and stored at −80 °C.

### Cytokine treatment for real-time PCR

For real-time PCR, HAEC and HUVEC were grown on 24-well plates to ~80 % confluence, serum-starved in collection medium for 16 h and then treated with 0.1 nM TNFα (PeproTech, Rocky Hill, NJ) in collection medium or ECPCM for 2 h at 37 °C, 5 % CO_2_. For some experiments, HAEC were co-treated with transforming growth factor (TGF)-β1 (PeproTech, Rocky Hill, NJ) and TNFα in collection medium for 2 h.

### RNA extraction, reverse transcription and real-time PCR

RNA samples were harvested from each well using the RNeasy Mini spin column kit (Qiagen, Hilden, Germany), according to the manufacturer’s instructions. Reverse transcription was performed using the QuantiTect RT kit (Qiagen, Hilden, Germany), according to the manufacturer’s instructions. Quantitative real-time PCR was performed using specific human TaqMan probes (Applied Biosystems, Foster City, CA) for E-selectin (Hs00950401_m1) and VCAM-1 (Hs01003369_m1). Human hypoxanthine-guanine phosphoribosyl transferase (HPRT1) TaqMan gene assay (4333768F) was used as the housekeeping gene. The experiments were performed using triplicate samples, and the data represent the average of at least three experiments ± standard error of the mean (SEM). All data from real-time PCR were normalized to the background control (collection medium alone) and expressed as fold-induction of gene expression relative to the control.

### Immunofluorescence microscopy

HAEC were grown on collagen-coated coverslips in 24-well plates at 37 °C, 5 % CO_2_ until confluent. Then, EC were serum-starved in collection medium for 16 h and treated at 37 °C, 5 % CO_2_ in collection medium or ECPCM with 0.1 nM TNFα for 10 or 30 min and 1 or 2 h. The following primary antibodies were used: rabbit anti-p65 (Cell Signalling, Danvers, MA) and sheep anti-PECAM-1 (R&D Systems, Abingdon, UK). AlexaFluor® 594 donkey anti-sheep and 488 donkey anti-rabbit (Invitrogen, Paisley, UK) were used as secondary antibodies.

### Protein extraction and western blot

Confluent EC were treated with 0.1 nM TNFα and proteins were extracted using radioimmunoprecipitation assay buffer (RIPA buffer; Sigma, St. Louis, MO) with protease and phosphatase inhibitors (Thermo Scientific, Loughborough, UK). SDS-PAGE was performed using a 10 % polyacrylamide gel and proteins were transferred onto a Hybond membrane. The primary antibodies mouse anti-β actin (Sigma, St. Louis, MO), rabbit anti-IkBα (AbCam, Cambridge, UK), rabbit anti-E-selectin, and anti-VCAM-1 (Santa Cruz Biotechnology, CA) were used, followed by appropriate secondary antibody conjugated to horseradish peroxidase (GE Healthcare, Little Chalfont, UK). Blots were developed using the GE Healthcare (Little Chalfont, UK) ECL+ System, according to the manufacturer’s instructions. Relative densitometric quantification of bands in western blots was performed using ImageJ software (https://imagej.nih.gov/ij/).

### U937 cell attachment assay

For the counting of adherent cells, HAEC were grown in 24-well plates as for immunofluorescent staining. When confluence was reached, the cells were serum-starved in collection medium for 16 h and then incubated in collection medium or ECPCM with or without 0.1 nM TNFα for 5 h at 37 °C, 5 % CO_2_. U937 cells (passage four to seven) were stained with 2 μM calcein AM (Invitrogen) and added on top of the cytokine-treated EC (300,000 cells/well) for 30 min at 37 °C, 5 % CO_2_. After the incubation period EC were washed with phosphate-buffered saline (PBS). Counting of the U937 cells attached to HAEC was performed using an Olympus BX51 microscope and a 10× objective: images of three non-overlapping fields of each well, on triplicate coverslips, were taken. The number of adherent U937 cells was determined using the automated cell counting software Image Pro Plus (Media Cybernetics). Variation in the number of cells was expressed as fold increase compared to the untreated control (collection medium). Differences between treatments were compared using GraphPad Prism software and one-way ANOVA followed by the post hoc Tukey test. The criterion for statistical significance was *p*-value <0.05.

### mRNA stability assay

HAEC at ~80 % confluence were serum-starved for 16 h in collection medium, pre-treated for 2 h with 0.1 nM TNFα in collection medium and then treated with collection medium or ECPCM with 0.1 nM TNFα and 3 μg/ml of actinomycin D (Sigma, St. Louis, MO) for an additional 30 min, 1, 2 or 4 h. Cells were processed for gene expression analysis by real-time PCR as described above.

### Chromatin immunoprecipitation (ChIP)

HUVEC were grown on 10 cm dishes until confluent and serum-starved in collection medium for 16 h before being treated with 0.1 nM TNFα in collection medium or ECPCM for 1 h at 37 °C, 5 % CO_2_. Cells were then processed for chromatin fixation and extraction using the ChIP-IT Express kit (Active Motif, La Hulpe, Belgium) according to the manufacturer’s instructions. Immunoprecipitation was performed using ~10 μg of DNA in all ChIP reactions and 4 μl of anti-p65 antibody (Active Motif, La Hulpe, Belgium) or isotype-matched control rabbit IgG (Cell Signalling, Danvers, MA). Samples were immunoprecipitated for 16–18 h at 4 °C. The obtained DNA was further purified using the Chromatin IP DNA Purification kit (Active Motif, La Hulpe, Belgium) and then used in real-time PCR. The primers and TaqMan probes used in the PCR reaction were designed by and purchased from Integrated DNA Technologies (Leuven, Belgium). Primers for E-selectin promoter: forward 5′-TTG TCC ACA TCC AGT AAA GAG G-3′, reverse 5′-AGG CAT GGA CAA AGG TGA AG-3′; probe 5′-/56-FAM/CCC CAA TGG CAT CCA AAA ACT TTC CC/36-TAMSp/-3′. Primers for VCAM-1 promoter: forward 5′-TTA ATA GTG GAA CTT GGCTGG G-3′, reverse 5′-GGA GTG AAA TAG AAA GTC TGT GC-3′; probe 5′-/56-FAM/TGT TGC AGA GGC GGA GGG AAA T/36-TAMSp/-3′. The immunoprecipitated DNA was quantified using a standard curve created with known concentrations of the input DNA. Binding of NF-kB to the specific promoter was calculated as fold-enrichment of the anti-p65 samples relative to the IgG control samples.

### In vivo experiments

JR5558 mice on a C57BL/6J background (five generations of backcross to C57BL/6J, Jackson Laboratory, Bar Harbor, ME) and kept as a homozygous line were used in these studies. These mice are not yet commercially available. The JR5558 mouse is an established model of spontaneous CNV, and has been used successfully for the identification and validation of a novel therapeutic target involving inflammation-driven CNV pathogenesis [19, 20]. Animals were kept on a 12-hour light–dark cycle and all experiments were conducted in accordance with Home Office guidelines (http://goo.gl/FLkirW) and the ARVO Statement for the Use of Animals in Ophthalmic and Vision Research (http://goo.gl/4LFOjD). Postnatal day (P) 21 mice were assessed for CNV lesions through fundus fluorescein angiography (FA) using a Kowa Genesis-Df fundus camera (Kowa, Sandhurst, UK). The animals received daily intraperitoneal administration of 0.5 ml of ECPCM or PBS for 7 days, from P22 to P28. Filter sterilized ECPCM or sterile buffer control was injected into the peritoneal cavity of the mice, without anesthesia, using standard 1 mL syringes equipped with 30G needles. At the age of P29 CNV lesions were analysed again by FA on masked samples. The area of the lesions was calculated on FA images using Photoshop software. On P30, mice were euthanized and eyes were harvested. Staining was performed using rabbit anti-PECAM-1 and rat anti-F4/80 primary antibodies (Abcam, Cambridge, UK). Macrophage quantification was performed by direct cell counting on masked samples: three CNV lesions were analysed on three separate eyes for each group, for a total of nine images for each treatment.

### Characterization of ECPCM

To calculate the IC50, confluent HAEC were serum-starved in collection medium for 16 h before treatment. ECPCM was serially diluted 1:2 in collection medium and tested, together with the collection medium control, on HAEC in the presence of 0.1 nM TNFα for 2 h at 37 °C, 5 % CO_2._ Cells were then processed for gene expression analysis by real-time PCR as described above. Relative E-selectin and VCAM-1 gene induction was expressed as percentage of control. GraphPad Prism software was used for non-linear regression analysis of the data for IC50 determination.

To determine if the active molecule was a protein 1 ml of ECPCM was heated at 95 °C for 15 min and approximately 0.2 units (5.5 mg) of proteinase K conjugated to agarose beads (Sigma, St. Louis, MO) were added, followed by incubation at 55 °C for 16 h. The enzyme was removed from the medium by centrifugation at 2660 × *g* for 10 min at 4 °C. For the RNase A/T_1_ (Fermentas, Sunderland, UK) treatment 1 ml of ECPCM was incubated with 20 μl of the enzyme mix at 37 °C for 1 h. After each treatment the ECPCM was cooled on ice and then stored at 4 °C until assayed.

The Griess reagent kit for nitrite determination (Invitrogen Paisley, UK) was used, according to manufacturer’s instructions, to determine nitric oxide (NO) levels in collection medium and ECPCM. ELISA kits for TGF-β1, interleukin (IL)-10, cyclic AMP (cAMP) (R&D Systems, Abingdon, UK) and prostaglandin I_2_ (PGI_2_) (MyBioSource, Upper Heyford, UK) were used, according to the manufacturer’s instructions, to determine the concentration of the molecules in collection medium and ECPCM.

### Coomassie blue polyacrylamide gel staining

Forty μl of proteinase K-treated or untreated ECPCM were mixed 1:5 with a reducing lane marker sample buffer (Thermo Scientific) and boiled at 95 °C for 5 min. Samples were loaded on a 7.5 % polyacrylamide gel for SDS-PAGE. The gel was then fixed for 30 min in 50:10:40 methanol:acetic acid:H_2_O and stained another 30 min in Coomassie blue working solution [concentrated Coomassie blue solution (2 g brillant blue in 50 ml methanol + 6 ml acetic acid) diluted 3:58 in 5:40:10 methanol:acetic acid:H_2_O]. De-staining was performed in 45:10:45 methanol:acetic acid:H_2_O until no background staining was observed.

### Statistics

GraphPad Prism software was used for all the statistical analysis. Multiple groups were compared using one-way ANOVA followed by the post hoc Tukey test, whilst comparison between two different groups was performed using the Mann-Whitney t-test. The criterion for statistical significance was *p*-value <0.05.

## Results

### ECPCM inhibits TNFα-induced expression of E-selectin and VCAM-1 in cultured HAEC

**Fig. 1:**
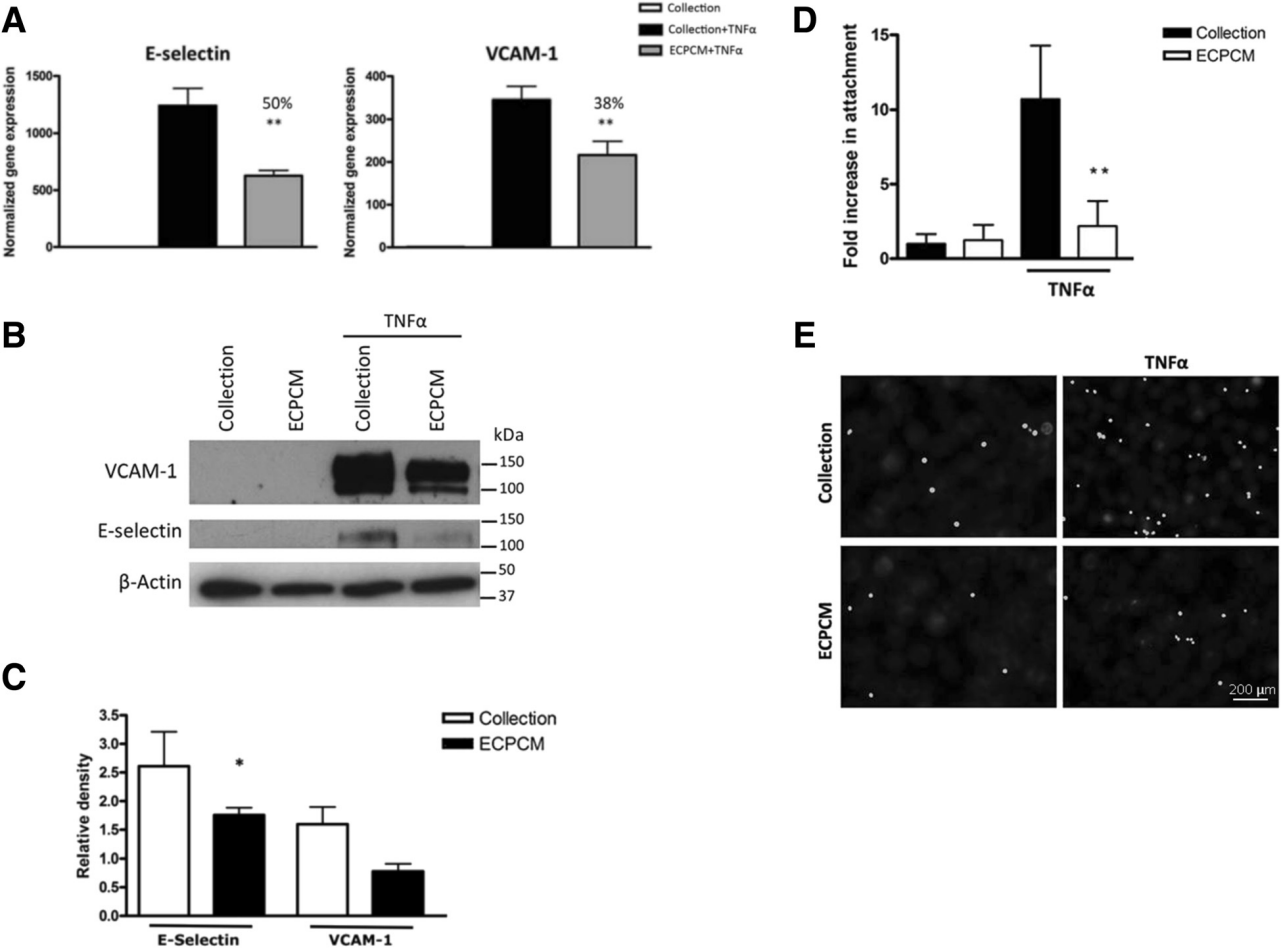
Inhibition of TNFα-induced expression of E-selectin and VCAM-1 by ECPCM. **a** HAEC were treated with TNFα for 2 h in collection medium or ECPCM, then relative gene expression levels normalized to the collection media control were determined by real-time PCR analysis. Percentage inhibition of gene expression by ECPCM was calculated by comparing treatment with ECPCM and TNFα to treatment with TNFα in collection medium. *p*-value: ** <0.01 compared to TNFα in collection medium. Data = mean ± SEM. **b** HAEC were treated for 6 h with or without TNFα in collection medium or ECPCM. Western blot analysis was performed with anti-VCAM-1, anti-E-selectin and anti-β-actin antibodies. The image is representative of three separate experiments. **c** Relative density quantification of western blot bands for TNFα-treated HAEC in panel (**b**), performed using ImageJ software. E-selectin and VCAM-1 bands were normalised to the β-actin loading control band. Data = mean ± SEM; *n* = 3 per treatment. *p*-value: * <0.05 compared to TNFα in collection medium. **d** U937 cells were stained with calcein AM and then placed on top of HAEC for 30 min. After three rinses with PBS, U937 attachment to EC was determined by counting the fluorescent cells. Changes in the number of attached cells are expressed as fold changes over the control (treatment in collection medium). *p*-value: ** <0.01 compared to TNFα in collection medium. Data = Mean ± SEM. **e** Representative images of U937 cells stained with calcein AM and attached to cytokine-activated HAEC treated in collection medium or ECPCM. Black background = HAEC; white dots = fluorescent U937 cells attached to the HAEC

TNFα is a potent inflammatory cytokine that induces expression of pro-inflammatory adhesion molecules such as E-selectin and VCAM-1 in EC [21–26]. Because the EC/matrix was efficacious in suppressing intimal hyperplasia in arteries in vivo, HAEC were deemed to be highly relevant primary cells and were used whenever possible in this investigation of the anti-inflammatory activity of ECPCM. As shown in Fig. 1a, ECPCM significantly inhibited the TNFα-induced gene expression of E-selectin and VCAM-1 in HAEC, by 50 and 38 %, respectively, compared to control. Significant inhibition of TNFα-induced expression of E-selectin by ECPCM was confirmed at the protein level (Fig. 1b-c). The biological effect of ECPCM was demonstrated through an attachment assay using the U937 cells, which are myeloid cells that adhere to the inflammation-activated endothelium. ECPCM strongly inhibited the adhesion of U937 cells to HAEC treated with TNFα (Fig. 1d-e).

### ECPCM does not affect NF-kB pathway activation

**Fig. 2:**
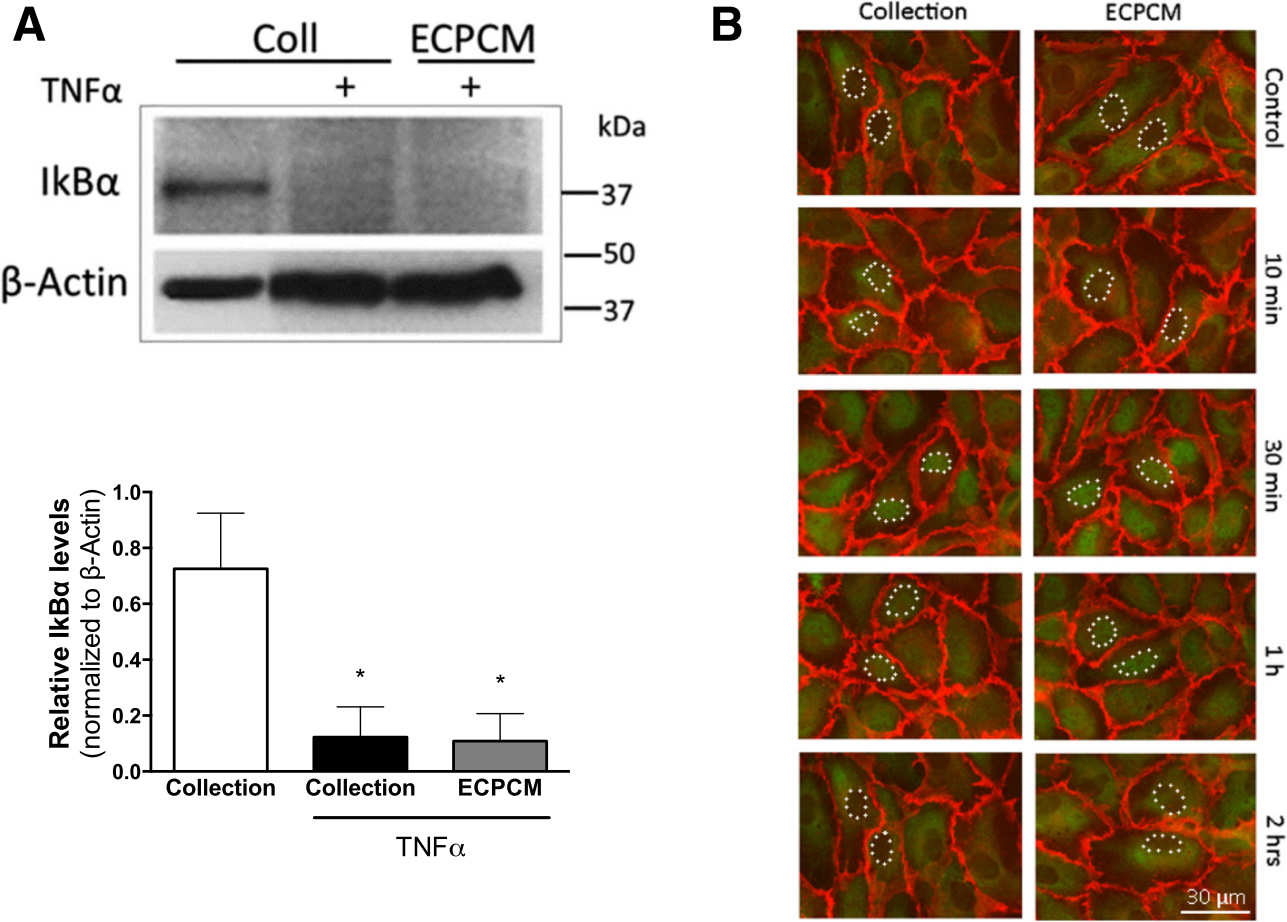
ECPCM does not affect TNFα-dependent activation of NF-kB or nuclear translocation of p65 in HAEC. **a** HAEC were treated for 30 min with collection medium or with TNFα in collection medium or ECPCM. Western blot analysis was performed using antibodies for β-actin and IkBα. TNFα induced IkBα degradation, and therefore NF-kB pathway activation, when collection medium or ECPCM was used during the treatment. The experiment was repeated at least three times with similar results, and representative blots are shown. *Bottom* panel, relative densitometric quantification of western blot bands for TNFα-treated HAEC. IκBα bands were normalised to the β-actin loading control band. Data = mean ± SEM; *n* = 5 per treatment. *p*-value: * <0.05 compared to collection medium control. **b** HAEC were treated with or without TNFα in collection medium or ECPCM for 10 min, 30 min, 1 h or 2 h. Immunofluorescence staining was performed with anti-PECAM-1 (*red*) and anti-p65 (*green*) antibodies. TNFα induced nuclear translocation of p65 upon treatment in both collection medium and ECPCM. Control: cells incubated in collection medium or ECPCM without TNFα for 2 h. Some nuclei are outlined with white dots to highlight the translocation of p65

TNFα-induced gene expression is typically mediated by activation of nuclear factor (NF)-kB, a transcription factor composed of the p65 and p50 subunits that is known for its important role in modulating the immune and inflammatory response. Activation of the pathway involves the phosphorylation and subsequent degradation of IkBα, the inhibitor that binds to NF-kB and retains it in the cytoplasm [27]. Western blots were performed to examine the protein expression levels of IkBα and immunofluorescence was used to determine the location of p65 in EC treated with TNFα in collection medium or ECPCM. Treatment with TNFα in collection medium induced IkBα degradation and NF-kB pathway activation with p65 translocation to the nucleus in HAEC, and ECPCM did not affect TNFα–dependent IkBα degradation or nuclear localisation of p65 (Fig. 2). These data suggest that the anti-inflammatory activity of ECPCM is not mediated by direct inhibition of NF-kB activation.

### ECPCM decreases TNFα-induced binding of the NF-kB p65 subunit to the E-selectin and VCAM-1 promoters

**Fig. 3:**
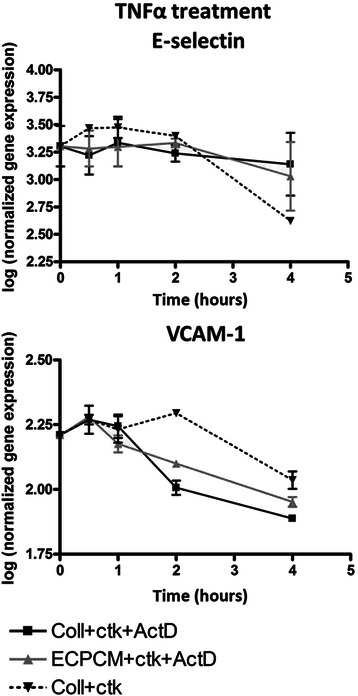
ECPCM treatment does not affect mRNA stability of E-selectin or VCAM-1 transcripts. HAEC were treated with TNFα in collection medium for 2 h. The medium was then changed to collection medium + TNFα (*dotted line*), collection medium + TNFα + actinomycin D (*black line*) or ECPCM + TNFα + actinomycin D (*grey line*). Transcript levels were assessed by real-time PCR after 30 min, 1, 2 and 4 h of treatment. The mRNA stability for both genes did not change upon ECPCM treatment. Data = mean ± SEM

Because ECPCM did not affect the activation and nuclear translocation of NF-kB, despite inhibiting TNFα-induced mRNA expression of E-selectin and VCAM-1, the effect of ECPCM on mRNA stability and transcriptional control of these genes was evaluated. Treatment of HAEC with ECPCM did not alter the mRNA stabilities of E-selectin or VCAM-1 (Fig. 3), indicating that the inhibition of gene expression is not mediated by modulation of mRNA stability.

**Fig. 4:**
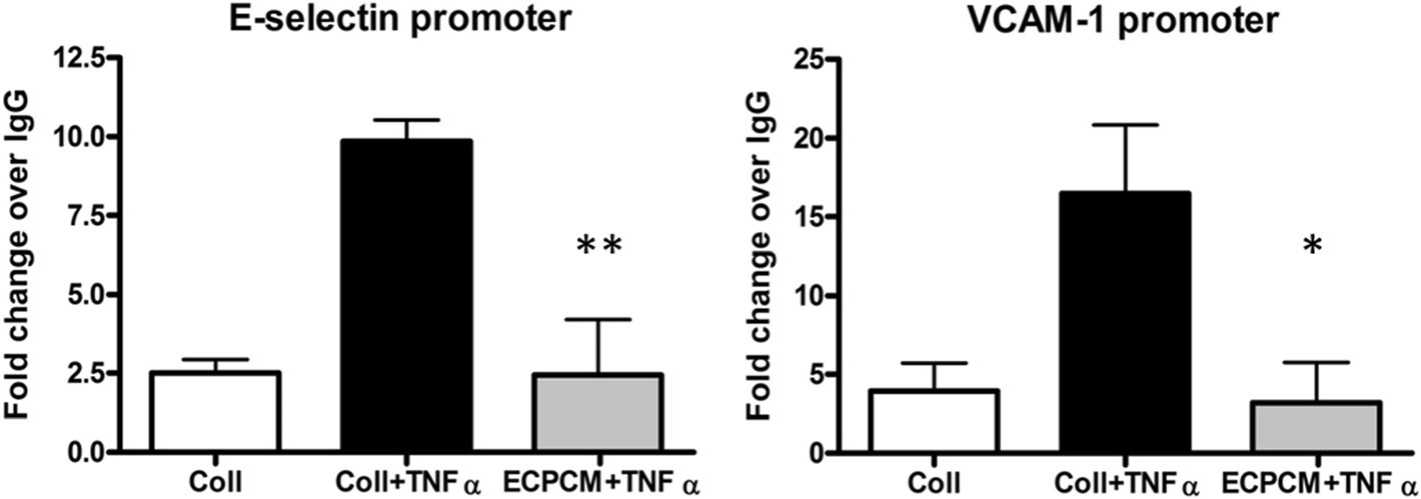
ECPCM significantly decreases binding of p65 to E-selectin and VCAM-1 promoters upon TNFα treatment. HUVEC were treated for 1 h with or without TNFα in collection medium or ECPCM. The extracted chromatin was immunoprecipitated using anti-p65 antibody or isotype-matched IgG as control. The immunoprecipitated DNA was then quantified using real-time PCR. The level of p65 binding to the specific promoters was expressed as fold-change over the IgG control. *p*-value: * <0.01, ** <0.001 comparing ECPCM + TNFα to collection medium + TNFα treatment. Data = mean ± SEM

To evaluate the effect of ECPCM on transcriptional control by NF-kB, chromatin immunoprecipitation (ChIP) experiments were performed to analyse TNFα-induced binding of NF-kB p65 to the E-selectin and VCAM-1 promoters. HUVEC were used for this assay due to the inability of HAEC to achieve the cell density required for adequate isolation of nuclei. In HUVEC, as in HAEC, ECPCM suppresses TNFα-induced expression of E-selectin and VCAM-1 without suppressing NF-kB activation and nuclear translocation (Additional file 1: Figure S1). ECPCM significantly suppressed TNFα-induced binding of p65 to the promoters of both E-selectin and VCAM-1 in HUVEC, to levels similar to those observed for the TNFα-free control (Fig. 4). These data suggest that ECPCM exerts its anti-inflammatory activity on TNFα mainly by inhibiting binding of NF-kB p65 to the promoters of target genes such as E-selectin and VCAM-1, thereby suppressing transcription of pro-inflammatory genes.

### ECPCM shows anti-inflammatory activity in an animal model of spontaneous CNV

To determine the therapeutic potential of ECPCM in vivo, a mouse model of inflammatory CNV was used. The JR5558 mouse is an established genetic model of spontaneous, multi-focal, bilateral CNV discovered as a spontaneous mutant line at the Jackson Laboratory [20]. These mice harbour a homozygous recessive mutation in an unknown gene(s) that leads to formation of subretinal neovascular tufts that originate from the choriocapillaris, starting between post-natal day P10 and P15. The CNV lesions are accompanied by macrophage infiltration and systemic depletion of monocytes and macrophages decreases lesion size without affecting lesion number, confirming that local inflammation and macrophage activity play a significant role in driving the growth of the CNV [6, 19, 20]. Fundus fluorescein angiography (FA) analysis was performed on JR5558 mice at P21 (day 0) to measure the baseline area of each CNV lesion. Animals were then treated for 7 days with intraperitoneal injection of ECPCM or buffer control. Lesion measurements by FA were repeated at P29 (day 8), and eyes were collected for immunostaining analysis at P30 (day 9).

**Fig. 5:**
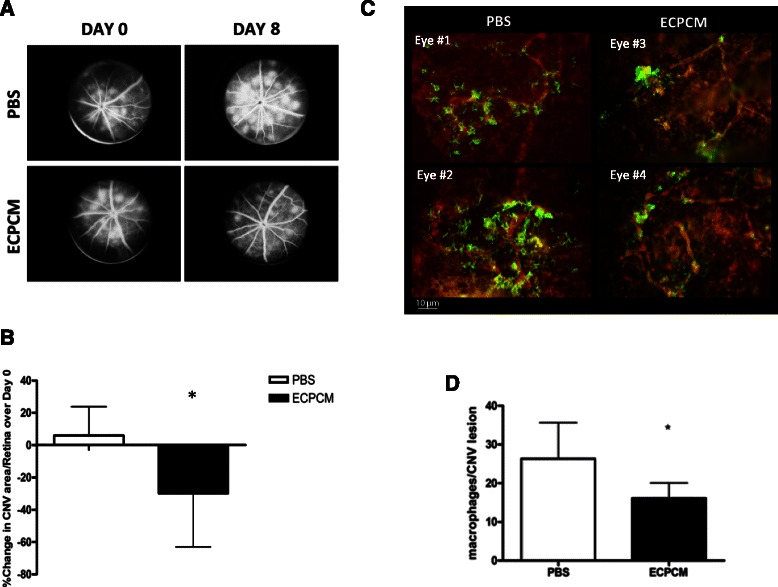
ECPCM reduces CNV area and macrophage recruitment to CNV lesions. JR5558 mice received daily intraperitoneal injections of ECPCM or PBS from P22 (day 1) to P28 (day 7). **a** Representative early phase fluorescein angiography images obtained from JR5558 mice before (P21, day 0) and after (P29, day 8) 7 days of treatment with either PBS or ECPCM. **b** CNV area/retina was determined at baseline (day 0) and after the treatment (day 8) using fluorescein angiography. The graph shows the % increase or decrease in CNV area/retina at day 8 compared to day 0 (+5.9 % for PBS and −29.89 % for ECPCM treatment). *p*-value: * <0.05. Data = mean ± SEM. **c** After 7 days of treatment with ECPCM or PBS, eyecups were collected and stained with anti-F4/80 antibody for activated macrophages (*green*) and anti-PECAM-1 antibody for EC (*red*) of the CNV. Two representative images for each group of treatment are shown. **d** Quantification of macrophage recruitment at CNV lesions expressed as macrophage number around each lesion. *p*-value: * <0.05. Data = mean ± SEM

Figure 5a-b shows that treatment with ECPCM for 7 days significantly reduced the area of CNV lesions per retina compared to the control treatment. Indeed, from day 0 (P21) to day 8 (P29) the average CNV area per retina increased by ~6 % in control mice, whereas it decreased by ~30 % on average in animals treated with ECPCM. No reduction in lesion number per retina was detected in any group (data not shown). Furthermore, macrophage recruitment to the CNV was reduced in animals treated with ECPCM compared to buffer control-treated mice (Fig. 5c-d). These results indicate the presence of at least one potent, soluble anti-inflammatory factor in the ECPCM.

### Characterisation of the anti-inflammatory activity

**Fig. 6:**
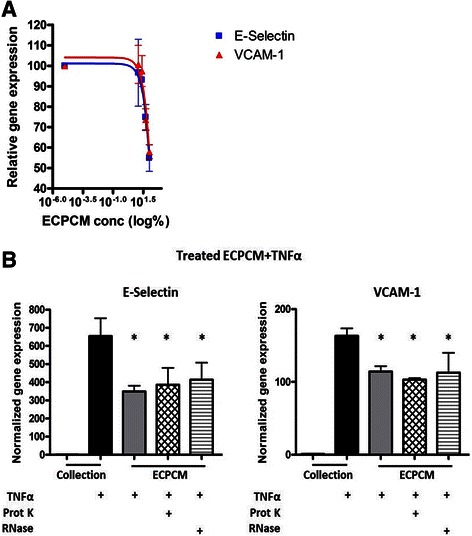
**a** Dose-dependent anti-inflammatory effects of ECPCM. HAEC were treated for 2 h with TNFα in collection medium, ECPCM, or serial dilutions (1:2) of ECPCM in collection medium. The graph shows the percentage of expression of E-selectin and VCAM-1 relative to that observed for TNFα in collection medium, as determined by real-time PCR analysis. Data = mean ± SEM. **b** ECPCM anti-inflammatory activity is not affected by proteinase K or RNase treatment. Gene expression of E-selectin and VCAM-1 was analysed by real-time PCR after treatment for 2 h with TNFα. HAEC were treated with collection medium, ECPCM or ECPCM previously treated with proteinase K or RNase. *p* value: * <0.001 compared to treatment with TNFα in collection medium. Data = mean ± SEM

To determine the IC50 of the anti-inflammatory activity, HAEC were treated with TNFα in serially diluted samples of ECPCM and levels of E-selectin and VCAM-1 mRNA were used as endpoints. ECPCM inhibited TNFα-induced expression of E-selectin and VCAM-1 in a dose-dependent manner, with an IC50 of 57 % ECPCM for E-selectin and 46 % ECPCM for VCAM-1 (Fig. 6a). A different lot of ECPCM was also tested in a separate experiment and produced similar results (data not shown). These data suggest that the anti-inflammatory activity in ECPCM is mediated by a soluble factor(s) that can be concentrated for further study.

It was next determined if the anti-inflammatory soluble factor in ECPCM might be a proteinase K-sensitive protein. ECPCM samples were incubated with proteinase K, a broad-spectrum serine protease, then separated on a polyacrylamide gel. Coomassie blue staining revealed substantial digestion of proteins in the sample (Additional file 2: Figure S2). The proteinase K-treated ECPCM significantly suppressed TNFα-induced expression of E-selectin and VCAM-1 in HAEC, with activity similar to that of the non-proteinase K-treated ECPCM (Fig. 6b). The anti-inflammatory factor in ECPCM is therefore not sensitive to proteinase K digestion and might not be a protein.

In recent years, it has been demonstrated that RNA molecules play important regulatory roles in cells. To determine if RNA molecules contribute to the anti-inflammatory effect, ECPCM was treated with a mix of RNase A/T_1_ exoribonucleases that degrade single strand RNA (ssRNA). In HAEC the RNase-treated ECPCM inhibited TNFα-stimulated expression of E-selectin and VCAM-1 to levels comparable to those observed with untreated ECPCM (Fig. 6b).

EC produce and release many molecules that control vascular physiology and homeostasis. Those with anti-inflammatory effects include NO [1–3], PGI_2_ [1–3, 28–30], TGF-β1 [31–34], IL-10 [35–40], and cAMP [41, 42]. To determine if any of these molecules were mediating the anti-inflammatory activity of ECPCM, their concentrations in the conditioned medium were compared to the levels detected in collection medium (Table 1). Levels of TGF-β1 were far greater in ECPCM than in the collection medium control. However, TGF-β1 is not resistant to proteinase K degradation, and collection medium supplemented with acid-activated TGF-β1 to a level higher to that observed in ECPCM (1000 pg/ml) failed to inhibit TNFα-induced expression of E-selectin and VCAM-1 (Additional file 3: Figure S3). Together these data suggest that TGF-β1 is not responsible for ECPCM anti-inflammatory activity. The levels of IL-10 and NO were lower in ECPCM than in collection medium, and levels of cAMP and PGI_2_ were similar between the samples tested. Based on these data, IL-10, cAMP, PGI_2_ and NO were also excluded as possible mediators of ECPCM anti-inflammatory activity.

**Table 1:** Average concentration of known anti-inflammatory molecules in collection medium and ECPCM

	Collection medium	ECPCM	Analysis concentration range	
			Min	Max
TGF-β1 (pg/ml)	61.81	749.4	0	2000
IL-10 (pg/ml)	77.62	44.96	0	500
cAMP (pmol/ml)	6.5	6.5	3.25	240
NO (μM)	21.72	0.072	0	50
PGI_2_ (ng/ml)	<2.5	<2.5	0	50

Overall, the results suggest that the soluble anti-inflammatory factor in ECPCM is not ssRNA; if it is a protein, it is proteinase K-resistant; and it is not any of the well-characterized anti-inflammatory molecules tested in our study.

## Discussion

EC are important producers of molecules that regulate vascular homeostasis. Although the nature and function of many EC-derived molecules have been identified over the past three decades, the EC secretome has by no means been completely defined. Here we offer some insight into a soluble anti-inflammatory factor released by EC in 3D culture in vitro and we demonstrate that systemic administration of this factor can reduce inflammation-associated CNV in vivo.

The NF-kB family of transcription factors plays an important role in mediating TNFα-induced expression of pro-inflammatory genes in EC [27, 43]. Soluble TNFα receptors are known to suppress pro-inflammatory activity, but it is unlikely that this is the mechanism employed by ECPCM, since ECPCM does not prevent TNFα-induced activation of NF-κB p65, nuclear translocation of p65, or turnover and degradation of IkBα in HAEC or HUVEC. Moreover, ECPCM does not affect the mRNA stability of E-selectin and VCAM-1. An explanation of the mechanism of action for ECPCM was provided by the ChIP experiments, which showed that ECPCM significantly reduces p65 binding to E-selectin and VCAM-1 promoters, thus inhibiting the transcription of these adhesion molecules upon TNFα stimulation in HUVEC. Binding of p65 to DNA is controlled by ubiquitination or acetylation of four lysine residues (Lys-122, −123, −314 and −315) [43]. Ubiquitination of p65 promotes its release from chromatin by targeting the protein for proteasomal degradation [43]. Since immunofluorescence analysis showed no detectable reduction in p65 levels upon ECPCM treatment, the degradation mechanism is unlikely to be involved. Acetylation of Lys-122 and −123 residues reduces p65 binding to DNA [44], and acetylated p65 is more easily exported to the cytoplasm [44]. Since immunostaining at different time points revealed no detectable difference in the nuclear translocation of p65 between ECPCM- and collection medium-treated EC, this mechanism can likely be excluded. Alternative mechanisms not excluded by our analysis include blocked acetylation at Lys-221 [45, 46], alkylation on Cys-38 [47, 48], reduced interaction between p65 and one or more of its many co-factors, or effects on the target DNA that prevent the binding of transcription factors to their target sequence on the promoter [49, 50].

The molecule responsible for ECPCM anti-inflammatory activity is resistant to proteinase K digestion and is not ssRNA-based. The anti-inflammatory activity might be mediated by a novel proteinase K-resistant protein or by double-stranded RNA. Double-stranded RNA in the form of microRNA (miRNA) alters expression of genes and proteins, primarily by negatively regulating post-transcriptional events via mRNA degradation and/or inhibition of translation [50]. Indeed, miRNA-10a regulates the pro-inflammatory phenotype of the endothelium through a post-transcriptional mechanism [51]. There is currently no mechanism to directly remove double-stranded RNA in solution, but since mRNA degradation of E-selectin and VCAM-1 was not affected by ECPCM, miRNA seems an unlikely candidate for mediating the anti-inflammatory activity.

Other methods used to characterize the anti-inflammatory factor(s) in ECPCM included heparin-agarose affinity purification, size-fractionation by column chromatography and dialysis cassettes. These techniques failed to generate useful information about the nature of the anti-inflammatory factor(s) because the anti-inflammatory activity of ECPCM was lost after each of these treatments (data not shown). It is unlikely that Gelfoam® alone mediated this anti-inflammatory activity, since the filtration process removed most if not all of the Gelfoam® used in the 3-D culture, but we cannot completely rule out a potential role for a soluble component released from the Gelfoam®. Work is on-going to identify the specific factor (or factors) that mediate the anti-inflammatory effects of ECPCM.

It is possible that the activity that we have identified in ECPCM maintains the EC in a non-inflammatory state under normal physiological conditions, promoting homeostasis and preventing excessive or uncontrolled EC activation. The anti-thrombotic state of the normal endothelium, for example, is maintained by a variety of molecules, both secreted and localized to the cell surface [1–3]. The anti-inflammatory activity we identified can act at distal sites and potentially contribute to systemic anti-inflammatory homeostasis of the vasculature. Interestingly, conditioned medium obtained from a similar EC/matrix culture was recently reported to have potent inhibitory effects on activation of dendritic cells and pro-inflammatory signalling associated with cancer progression [52, 53]. Collagen matrix culture may promote anti-inflammatory activity in other epithelia as well. Bronchial epithelial cells cultured in a similar 3-D collagen matrix produced bioactive compounds that facilitated airway repair, at least in part by reducing tissue inflammation [54].

We have demonstrated the anti-inflammatory effects of ECPCM in an animal model of spontaneous CNV associated with and driven by inflammation [19, 20]. CNV is a major blinding complication associated with AMD and is characterised by pathological inflammation and angiogenesis [6, 11]. The reduction in macrophage recruitment and associated CNV lesion size following treatment with ECPCM is particularly promising given that intraperitoneal delivery reduced local inflammatory reactions in the eye and also that only a small volume of unconcentrated ECPCM was sufficient for this response. The anti-CNV effect of ECPCM is consistent with the pre-clinical observation that EC/matrix formulation surgically placed perivascularly significantly reduced IH as well as leukocyte recruitment to the injured vessels in animal models [16]. The EC/matrix formulation (Vascugel®) is currently in phase II clinical trials for treating IH associated with arteriovenous access and graft failure in patients with end-stage renal disease (http://www.shiretrials.com/studies/). Our data confirm the potent anti-inflammatory effect of this therapy and support further characterization and identification of this novel activity in ECPCM, as well as its translational research for treating vascular inflammation associated with various pathologies. Our findings also provide new insights into EC biology and confirm the prominent role of EC-derived factors in regulating vascular homeostasis.

## Conclusions

Our study showed that conditioned medium from EC grown on collagen particles has a potent anti-inflammatory activity, both in vitro and in vivo, which is mediated by the inhibition of the binding by the activated transcription factors to the promoters of pro-inflammatory genes. These results provide a novel potential therapeutic strategy for the treatment of vascular inflammation and its associated pathologies.

## Additional files

Additional file 1: Figure S1. In HUVEC, as in HAEC, ECPCM inhibits TNFα-induced expression of E-selectin and VCAM-1 without affecting TNFα-dependent activation of NF-kB and nuclear translocation of p65. (**A**) HUVEC were treated with TNFα for 2 h in collection medium or ECPCM, then relative gene expression levels were determined by real-time PCR analysis and normalized to the collection medium control. Percentage inhibition of gene expression by ECPCM was calculated by comparing treatment with ECPCM plus TNFα to treatment with TNFα in collection medium. *p*-value: *** <0.001 compared to TNFα in collection medium. Data = mean ± SEM. (**B**) HUVEC were treated with or without TNFα in collection medium or ECPCM for 10 min, 30 min, 1 h or 2 h. Immunofluorescence staining was performed using anti-PECAM-1 (red) and anti-p65 (green) antibodies. TNFα induced nuclear translocation of p65 in both collection medium and ECPCM. Control: cells incubated in collection medium or ECPCM without TNFα for 2 h. Some nuclei of the cells are outlined with white dots to highlight the translocation of p65. Interestingly, activation of NF-kB p65 by TNFα occurred more rapidly and lasted longer in HUVEC compared to HAEC (**C**) HUVEC were treated for 30 min with collection medium or with TNFα in collection medium or ECPCM. Western blot analysis was performed using antibodies to β-actin and IkBα. The experiment was repeated at least three times with similar results. Representative blots are shown. Right panel, relative densitometric quantification of western blot bands for TNFα-treated HUVEC, performed using ImageJ software. IκBα bands were normalised to the β-actin loading control band. Data = mean ± SEM; *n* = 4 per treatment. *p*-value: *** <0.001 compared to collection medium control. (PDF 1529 kb) Additional file 2: Figure S2. Coomassie blue staining of untreated- and proteinase K-treated ECPCM. The band of ~65 kDa in the ECPCM lane corresponds to serum albumin. Absence of this band in the proteinase K-treated ECPCM lane confirmed substantial protein digestion. This experiment was repeated three times with similar results; representative images are shown. (PDF 51 kb) Additional file 3: Figure S3. TGF-β1 effects on cytokine-induced expression of E-selectin and VCAM-1. HAEC were treated for 2 h with 0.1 nM TNFα in ECPCM, collection medium or collection medium with increasing amounts of TGF-β1. Relative gene expression levels were determined by real-time PCR analysis and normalized to the collection medium control. *p*-value: * <0.05 compared to treatment in collection medium with TNFα. Data = mean ± SEM. (PDF 230 kb)

## Abbreviations

ChIP: chromatin immunoprecipitation
CNV: choroidal neovascularisation
Coll: collection medium
Ctk: cytokine
EC: endothelial cells
ECP: endothelial cells grown on collagen particles
ECPCM: endothelial cells on collagen particles-conditioned medium
HAEC: human aortic endothelial cells
HUVEC: human umbilical vein endothelial cells
NF-kB: nuclear factor-kB
TNFα: tumor necrosis factor alpha
VCAM-1: vascular cell adhesion molecule-1
