# Supplementary figures and images for: A novel endothelial-derived anti-inflammatory activity significantly inhibits spontaneous choroidal neovascularisation in a mouse model
Source: Vasc Cell. 2016 May 11;8:2. doi: 10.1186/s13221-016-0036-4 (PMC4864930; doi:10.1186/s13221-016-0036-4)

Figure S1

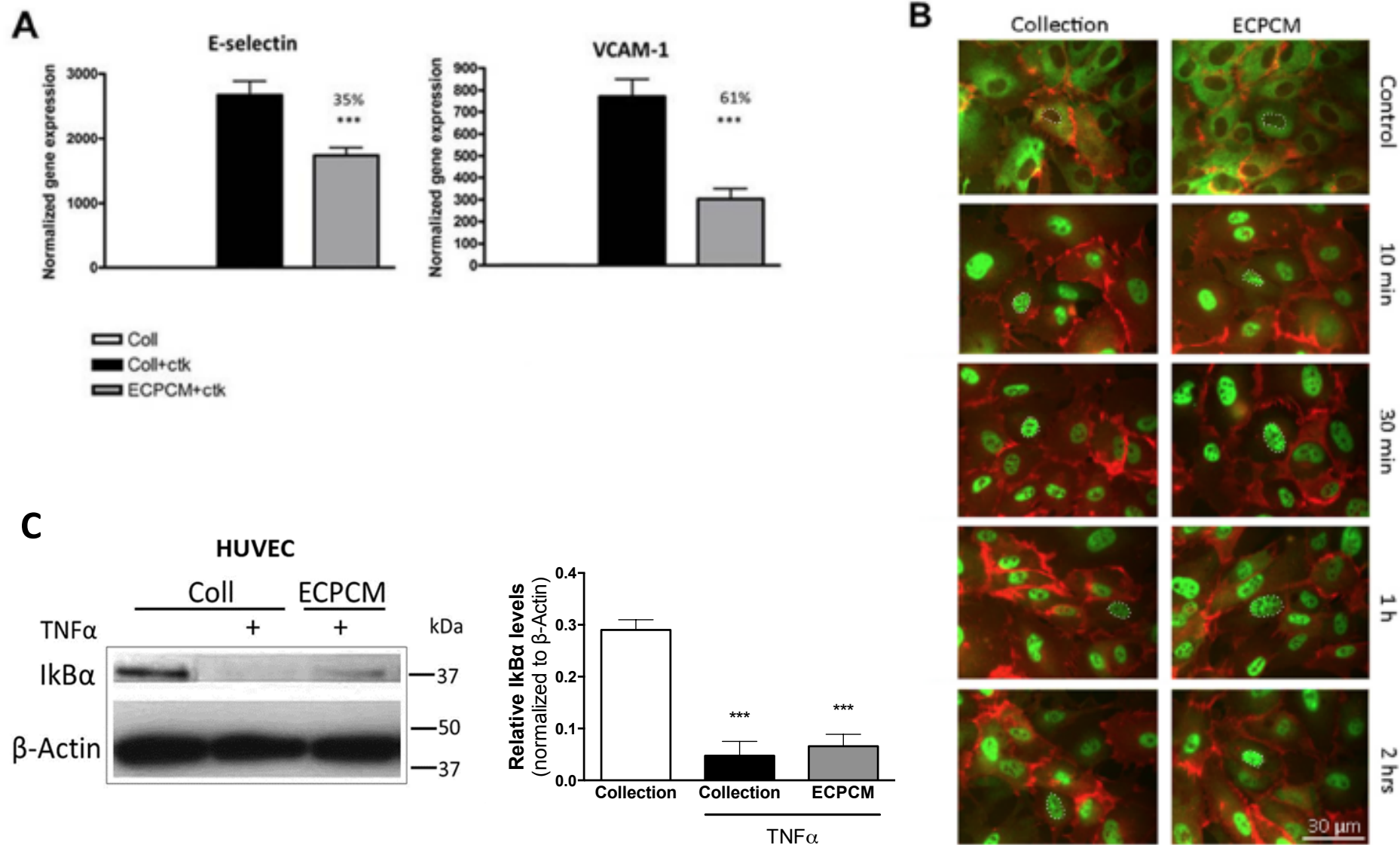

Supplement: Additional file 1: Figure S1. — In HUVEC, as in HAEC, ECPCM inhibits TNFα-induced expression of E-selectin and VCAM-1 without affecting TNFα-dependent activation of NF-kB and nuclear translocation of p65. (A) HUVEC were treated with TNFα for 2 h in collection medium or ECPCM, then relative gene expression levels were determined by real-time PCR analysis and normalized to the collection medium control. Percentage inhibition of gene expression by ECPCM was calculated by comparing treatment with ECPCM plus TNFα to treatment with TNFα in collection medium. p-value: *** <0.001 compared to TNFα in collection medium. Data = mean ± SEM. (B) HUVEC were treated with or without TNFα in collection medium or ECPCM for 10 min, 30 min, 1 h or 2 h. Immunofluorescence staining was performed using anti-PECAM-1 (red) and anti-p65 (green) antibodies. TNFα induced nuclear translocation of p65 in both collection medium and ECPCM. Control: cells incubated in collection medium or ECPCM without TNFα for 2 h. Some nuclei of the cells are outlined with white dots to highlight the translocation of p65. Interestingly, activation of NF-kB p65 by TNFα occurred more rapidly and lasted longer in HUVEC compared to HAEC (C) HUVEC were treated for 30 min with collection medium or with TNFα in collection medium or ECPCM. Western blot analysis was performed using antibodies to β-actin and IkBα. The experiment was repeated at least three times with similar results. Representative blots are shown. Right panel, relative densitometric quantification of western blot bands for TNFα-treated HUVEC, performed using ImageJ software. IκBα bands were normalised to the β-actin loading control band. Data = mean ± SEM; n = 4 per treatment. p-value: *** <0.001 compared to collection medium control. (PDF 1529 kb) [file 13221_2016_36_MOESM1_ESM.pdf]

Figure S2

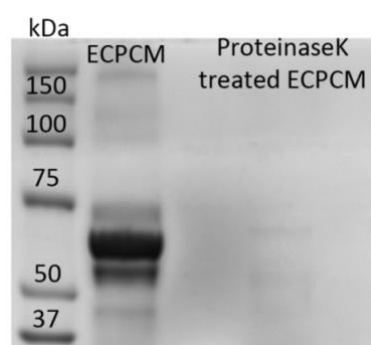

Supplement: Additional file 2: Figure S2. — Coomassie blue staining of untreated- and proteinase K-treated ECPCM. The band of ~65 kDa in the ECPCM lane corresponds to serum albumin. Absence of this band in the proteinase K-treated ECPCM lane confirmed substantial protein digestion. This experiment was repeated three times with similar results; representative images are shown. (PDF 51 kb) [file 13221_2016_36_MOESM2_ESM.pdf]

Figure S3

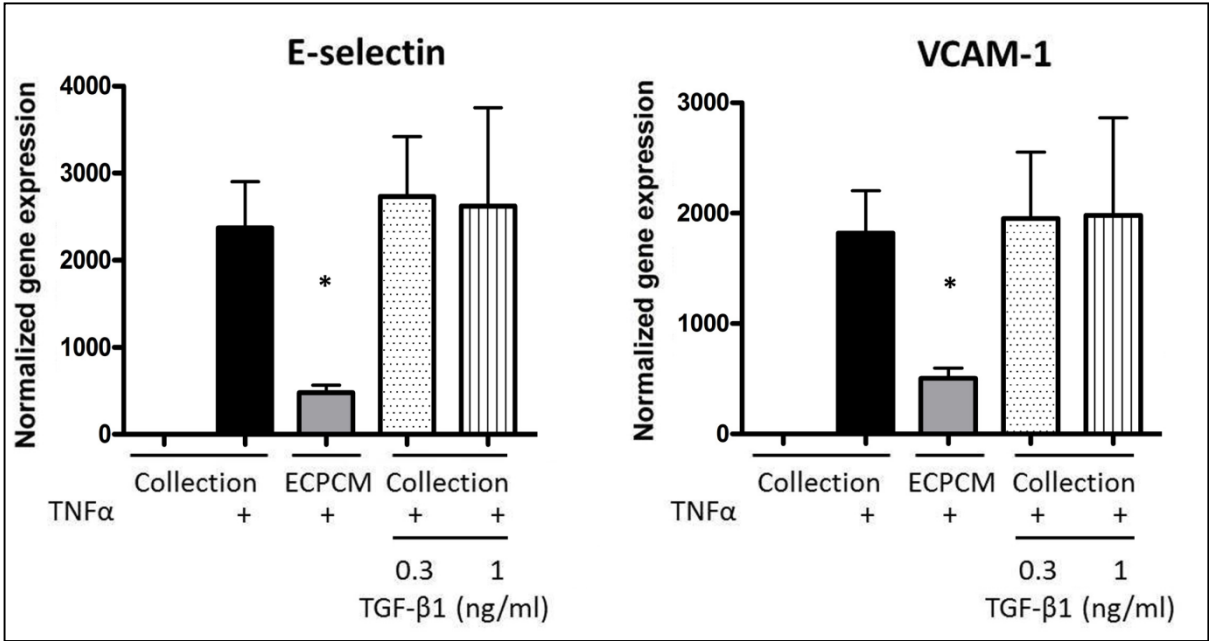

Supplement: Additional file 3: Figure S3. — TGF-β1 effects on cytokine-induced expression of E-selectin and VCAM-1. HAEC were treated for 2 h with 0.1 nM TNFα in ECPCM, collection medium or collection medium with increasing amounts of TGF-β1. Relative gene expression levels were determined by real-time PCR analysis and normalized to the collection medium control. p-value: * <0.05 compared to treatment in collection medium with TNFα. Data = mean ± SEM. (PDF 230 kb) [file 13221_2016_36_MOESM3_ESM.pdf]
